# Supplementary material for: The antioxidant system response to drought-stressed Diospyros lotus treated with exogenous melatonin
Source: PeerJ. 2022 Sep 19;10:e13936. doi: 10.7717/peerj.13936 (PMC9496507; doi:10.7717/peerj.13936)
Supplement: Supplemental Information 1 [file peerj-10-13936-s001.doc]

**Table S1 Composition of Hoagland nutrient solution.**

|  | Formula | Molecular weight  g·mol^-1^ | Concentration of stock solution (mM) | Concentration of stock solution (g·L^-1^) | Per L nutrient solution  (mL) |
| --- | --- | --- | --- | --- | --- |
| Macronutrients | KNO_3_ | 101.10 | 1000 | 101.10 | 6.0 |
|  | Ca(NO_3_)_2_·4H_2_O | 236.16 | 1000 | 236.16 | 4.0 |
|  | NH_4_H_2_PO_4_ | 115.08 | 1000 | 115.08 | 2.0 |
|  | MgSO_4_·7H_2_O | 246.48 | 1000 | 246.49 | 1.0 |
| Micronutrients | KCL | 74.55 | 25 | 1.864 | 2.0 |
|  | H_3_BO_3_ | 61.83 | 12.5 | 0.773 | 2.0 |
|  | MnSO_4_·H_2_O | 169.01 | 1.0 | 0.169 | 2.0 |
|  | ZnSO_4_·H_2_O | 287.54 | 1.0 | 0.288 | 2.0 |
|  | CuSO_4_·5H2O | 249.68 | 0.25 | 0.062 | 2.0 |
|  | NaMoO_4_·2H_2_O | 277.95 | 0.25 | 0.0695 | 2.0 |
|  | EDTA-FeNa·3H_2_O | 421.09 | 53.7 | 26.9497 | 0.3-1.0 |
