# Supplementary material for: The antioxidant system response to drought-stressed Diospyros lotus treated with exogenous melatonin
Source: PeerJ. 2022 Sep 19;10:e13936. doi: 10.7717/peerj.13936 (PMC9496507; doi:10.7717/peerj.13936)
Supplement: Supplemental Information 2 [file peerj-10-13936-s002.docx]

Table S2 Primer sequences (5’ → 3’) for qRT-PCR.

| Gene names | Primer sequence (5’-3’) |
| --- | --- |
| β-actin-F | CATGGAGAAAATCTGGCATCATAC |
| β -actin-R | GAAGCACTGGGTGCTCTTCTG |
| *Dl-SOD -*F | ATATTAAAATATATCATAATGACTA |
| *Dl-SOD* -R | CACCACAAGCAAGACGACCA |
| *Dl-CAT*-F | CCCAACCCCAAGTCTCACA |
| *Dl-CAT* -R | GGAACACCCAAATCATCGAA |
| *Dl-POD*-F | GAGAAAGAAATAGAAAGAGAGAGAG |
| *Dl-POD* -R | CAGCACAGAGCCATCACAAC |
| *Dl-RBOH-A*-F | ATCACCTGTTATGTTCCGCTTCCG |
| *Dl-RBOH -A*-R | CTTGCTTCCTCGCTCACTCTTCG |
